# Pilot study of an app-supported psychosocial prevention intervention: a mixed-methods approach

**DOI:** 10.1186/s40814-025-01737-y

**Published:** 2025-12-01

**Authors:** Jan Gehrmann, Johannes Stephan, Jana Dehner, Ananda Stullich, Matthias Richter, PE³PP study group, Olaf Ballaschke, Florian Finke, Frank Gabel, Anastasia Grossmann, Claudia Gruhner, Kathleen Kaminski, Andreas Seemann

**Affiliations:** 1 Chair of Social Determinants of Health, Department of Health and Sport Sciences, TUM School of Medicine and Health, Technical University of Munich, Munich, Germany; 2 Institute of General Practice and Health Services Research, Department of Clinical Medicine, TUM School of Medicine and Health, Technical University of Munich, Munich, Germany

**Keywords:** Mental health, Digital health, Mixed methods, Prevention, Pilot study, Medical apps

## Abstract

**Background:**

Work-related health issues, particularly those affecting mental health, are increasing, leading to significant costs for health systems and impacting individuals’ ability to work. Preventive interventions using digital health applications promise to mitigate these issues by promoting mental well-being and reducing risk factors.

**Objective:**

The study aimed to pilot the new prevention intervention “RV Fit Mental Health” which combines an intensive inpatient phase with an app-supported digital outpatient phase. The primary objectives were to understand the target group, evaluate the acceptability and feasibility of the intervention, and identify areas for optimization based on participant feedback.

**Methods:**

A mixed-methods approach was employed, combining qualitative and quantitative data. Qualitative data were gathered from 18 participants through focus groups. Quantitative data were collected from 21 participants using various validated instruments, including the Work Ability Index (WAI), World Health Organization Quality of Life-BREF (WHOQOL-BREF), Depression Anxiety Stress Scale-21 (DASS-21), and eHealth Literacy scales. Data analysis was conducted using descriptive statistics and content analysis.

**Results:**

Qualitative results highlighted the acceptability of the intervention and identified several challenges, including the transition between the different phases of the intervention and the diversity of participants’ backgrounds and health conditions. Participants appreciated the holistic approach of combining an inpatient and app-supported outpatient phase but suggested further adjustments to enhance usability and integration into daily life. Quantitative findings indicated a heterogeneous group of participants.

**Conclusions:**

The “RV Fit Mental Health” intervention shows promise for addressing work-related mental health issues. The initial results of the pilot study indicate feasibility and good acceptance of the intervention. However, further refinement and a larger-scale evaluation are necessary to confirm its efficacy and optimize its implementation. If successful, this approach could strengthen participation in working life and reduce mental health-related work incapacity.

**Trial registration:**

DRKS-ID: DRKS000308188 (https://drks.de/search/de/trial/DRKS00030818)

**Supplementary Information:**

The online version contains supplementary material available at 10.1186/s40814-025-01737-y.

## Key messages regarding feasibility

- The novelty of the new intervention "RV Fit Mental Health" as a prevention intervention raised uncertainties about its suitability and acceptability for a heterogeneous group of participants with diverse diagnoses and varying stages of illness, particularly in balancing early prevention goals with the needs of those experiencing more advanced mental health challenges, as well as managing the transition between the intensive inpatient and digital outpatient phases.
- Key feasibility findings included high acceptability of the intervention, with participants reporting positive behavioral changes and valuing the continuity provided by the digital phase, although varying digital literacy levels influenced engagement and adherence.
- Implications for the main study include tailoring digital components to participants´ literacy levels, enhancing the transition between phases, and ensuring modules are individualized to support diverse health needs.

## Background

### Work-related health issues and the potential of mental health prevention and digital health

Continuous and excessive stress related to work can impact individuals’ physical and psychological health. Work-related health issues leading to incapacity for work have been increasing worldwide [1–5] and can develop into burn-out syndrome and increase the risk of various mental disorders [6–8]. For example, several studies in Germany commissioned by health insurance companies reported an increasing number of sick days due to mental health issues [9, 10]. The German Pension Insurance Association—responsible for disability pension—also reported a significant increase due to mental health problems [11]. Long-term incapacity for work due to mental health issues also leads to significant costs for the health systems [4].

Preventive efforts based on evidence-based interventions offer great potential to reduce risk factors and initiate positive behavioral change [6, 9, 12, 13]. Recently, there has been a growing emphasis on promoting well-being and positive mental health to prevent mental disorders, as poor mental health has significant social and economic impacts by reducing mental illness-related morbidity at both societal and individual levels [14–16]. Effective prevention interventions bolster environmental and individual resources while minimizing risk factors [6, 9, 14].

Digital health is a promising approach to the prevention of mental health risks, as positive results have been achieved so far by using elements such as psychoeducation, therapeutic support, or the improvement of health literacy embedded within digital health approaches [17–20]. Digital health applications can be used flexibly in terms of time and location, allowing them to be integrated into everyday life alongside working life [18–20]. The use of digital health applications is influenced by various determinants that affect their effective and efficient use, ranging from environmental and social factors to motivational factors or the accessibility of technological devices in the sense of digital inclusion [21–27]. Emphasized in the discussion around digital health applications is the need for and importance of analyzing the acceptance and needs of the groups who will be using the digital health applications, especially regarding the question of successful implementation [28–35].

In the PE^3^PP project (“Development, piloting, and evaluation of an app-supported psychosocial prevention intervention”), a new intervention called “RV Fit Mental Health” was developed to promote and strengthen participation in work life [23].

### Objectives of this study

This study examined the piloting of the newly developed prevention intervention “RV Fit Mental Health.” We aim to answer the following research questions: (1) Characterize and understand the target group, (2) examine and understand the acceptability and feasibility of the app-supported intervention regarding its specifics and challenges, and (3) explore possible adjustments and optimization of the intervention based on the participants’ experiences, needs, and perspectives.

## Methods

### Study background

The project “Development, piloting, and evaluation of an app-supported psychosocial prevention intervention” (German acronym PE^3^PP) runs from October 2021 until September 2026 and is led by the German Pension Insurance Central Germany. The prevention intervention called “RV FIT Mental Health” aims to promote and strengthen individuals’ participation in work life. It is a prospective multicentric study. The two central German rehabilitation clinics, Median Klinik Bad Gottleuba and SRH Medinet Burgenlandklinik, led the development of the intervention and are conducting it. A further PE^3^PP project partner is the health insurance company Allgemeine Ortskrankenkasse PLUS (AOK PLUS) in Saxony and Thuringia. Nine-hundred sixty participants are expected to undergo the intervention overall from September 2023 to September 2026. As a project partner, the Chair of Social Determinants of Health at Technical University of Munich (TUM) is responsible for the scientific accompaniment and evaluation of the project. Ethical approval was obtained from the Ethics Committee at the TUM School of Medicine and Health (2022-523 S-SR; 2023-316 S-SB).

### Study intervention

The intervention lasts 14 weeks. It begins with a 2-week inpatient phase in one of the two clinics and continues with a 12-week app-supported training phase. The participants complete the inpatient intervention in small groups of up to 10 participants per clinic. During the inpatient and outpatient phases, participants receive intensive therapeutic support. The intervention targets explicitly mental health issues connected to or worsened by the work environment, including affective disorders, phobic and other anxiety disorders, adjustment disorders, somatoform disorders, and burnout. The range of ICD diagnosis thus includes the following: F30–39 (affective disorders), F40.0–40.9 (phobic disorders), F41.0–41.9 (any anxiety disorders), F43.2 (adaption disorder), F45.0–45.9 (somatoform disorders), and Z73 (burnout).

The conceptual development of the intervention was informed by established psychological and behavioral frameworks commonly applied in preventive mental health research. Although no single theoretical model was formally defined, the intervention follows an evidence-based rationale integrating cognitive-behavioral approaches focusing on stress management, emotion regulation, and psychoeducation[36–40]. Moreover, it conceptually draws on Bandura’s social cognitive theory, particularly the notions of self-efficacy and self-regulation, as well as Antonovsky’s salutogenic model emphasizing resource orientation and the sense of coherence as protective factors for mental health[41–44]. Together, these frameworks provide a robust theoretical and empirical foundation guiding the structure, mechanisms of change, and intended preventive outcomes of the intervention.

The intervention covers psychosocial elements like psychological stabilization, self-care, and “first aid” for issues such as burnout, depression, anxiety, sleep disorders, and pain. It includes training in social and emotional skills, professional and personal stress management, interaction and communication training, and mindfulness-based and body-oriented therapy elements. The outpatient training phase is based on an app (MindDistrict) that establishes a digital connection between the clinic and the participants. The training plan integrated into the app contains various modules that can be used during this phase. The app offers various methods and formats for conveying content, such as exercises, instructions, training courses, and seminars in video and audio formats. In addition, interaction and communication functions such as chats and video calls enable exchange within the app. Thus, the app supports and accompanies the transfer of knowledge and behavior to the participants’ everyday lives. Furthermore, during the training phase, each participant has an individual coaching session with their therapeutic support person every 4 weeks. An overview of the modules, the didactical realization of the intervention, and the comprehensive scientific evaluation can be found in the study protocol [23].

### Study design

This study outlines the piloting of the prevention intervention, which took place between September 2023 and March 2024.

The piloting was evaluated using a convergent mixed-methods approach with a strong focus on qualitative methods [23, 45–47]. The qualitative research strand is weighted more heavily in the pilot phase, while in the subsequent evaluation the weighting is in favor of the quantitative research strand. Qualitative data was collected using focus groups with the participants. Data collection occurred in parallel: quantitative data were collected within the first 2 days of the inpatient phase, while qualitative focus groups took place at the end of the inpatient phase as well as during the digital phase. Data triangulation as a methodological approach enhances the validity of results by integrating qualitative and quantitative data. This process allows for a more comprehensive analysis by capturing multiple perspectives and reinforcing findings through subsequent data collection and analysis which is particularly valuable in evaluation and implementation research, as it facilitates a dynamic understanding of intervention outcomes and contextual influences [23, 48–51]. The overall analytical approach is presented in the study protocol [23]. The aim of this study was to identify opportunities, challenges, barriers, and optimization possibilities for the intervention. Furthermore, sociodemographic variables and various outcomes such as workability, quality of life, and stress were analyzed descriptively to characterize the sample and test the feasibility of quantitative data collection and the plausibility of the data. To systematically assess the feasibility of the intervention, we conceptualized feasibility as the extent to which the intervention procedures could be implemented as intended and integrated into the clinical setting. Feasibility was examined through indicators related to (1) process feasibility (recruitment, participation, and retention rates), (2) data feasibility (completeness and plausibility of quantitative assessments), and (3) implementation feasibility (participant engagement, app usage, and acceptability reported in focus groups). Specific metrics such as recruitment success, attendance, and completeness of data entry were collected to gain initial insights into the feasibility of implementation. In the “Results” section, these dimensions are reported separately to distinguish between the feasibility of study procedures and the feasibility of the intervention delivery as experienced by participants. Overall, no progression criteria were deliberately established due to the multiphase structure of the project, which includes development, piloting, and subsequent evaluation of the intervention. As this was a pilot study, the primary objective was to examine feasibility and implementation rather than to assess effectiveness. Given the small sample size, a pre-post design would not have provided meaningful statistical power. Accordingly, this phase focused on qualitative insights, baseline assessments of participants’ burden profiles, and the exploration of both data collection and intervention feasibility. The evaluation of effectiveness was intentionally deferred to the subsequent large-scale evaluation phase of the project.

We followed the guidelines for reporting non-randomized pilot and feasibility studies and used the Consolidated Standards of Reporting Trials (CONSORT) to report the pilot study [52, 53]. Reporting the qualitative data, we followed the COnsolidated criteria for REporting Qualitative research (COREQ) [54].

### Sample and recruitment

The German Pension Insurance Central Germany contacted eligible insureds and proactively suggested participation in the “RV Fit Mental Health” prevention intervention. The suggestion for participation in the intervention was based on rejected rehabilitation applications. People submitted an application for medical psychosomatic rehabilitation. If the application for rehabilitation is rejected, people with specific ICD diagnoses in the F area, which emerge from the rehabilitation application, are offered the “RV Fit Mental Health” prevention intervention by the German Pension Insurance Central Germany. This was part of a proactive effort to identify individuals who might benefit from the intervention and meet the eligible criteria: rejected medical psychosomatic rehabilitation applications with corresponding diagnoses, insured in the German Pension Insurance Central Germany, and corresponding ICD diagnoses (see section “Study intervention”). In the piloting stage, presented in this study, 27 people participated in the intervention. We have no information regarding non-participation.

In the qualitative data collection, no specific target sample size was set for the pilot study; recruitment aimed to achieve a sample large enough to assess feasibility and acceptability and guarantee theoretical saturation of the findings. In the quantitative data collection, we aimed for a full survey of all participants. The sample size is not based on feasibility outcomes. Nevertheless, key metrics were collected to gain initial insights into implementation feasibility, which will be further examined in the main study. These metrics included the number of recruited and participating individuals, completion rates of the quantitative survey, and qualitative feedback on usability and engagement with the app.

#### Qualitative sample

Four focus groups (2 in each clinic) with 6 to 10 participants were planned during the piloting. We used a random sampling method. Contact with the possible participants was initiated via the respective clinical staff. If participants were interested, the clinics forwarded the date and number of participants. Focus groups (FG) were planned equally in presence in the inpatient phase (two FG) as well as digitally in the outpatient phase (two FG). Inclusion criteria are participation in the prevention intervention and sufficient German language skills.

#### Quantitative sample

We aimed to survey all participants who took part in the inpatient phase of the RV Fit Mental Health intervention between October and December 2023, which corresponds to a non-probability sample. On the flyer handed out by the clinical staff are instructions and online access for the quantitative data collection. Inclusion criteria are participation in the prevention intervention and sufficient German language skills.

### Sample size justification

As this was a pilot study, no formal sample size calculation was conducted. Following established methodological guidance for feasibility and pilot studies [55, 56], the sample size was determined pragmatically to assess feasibility, acceptability, and implementation aspects rather than to achieve statistical power or test hypotheses. The sample size was based on the practical capacity of the participating clinics (a theoretical maximum of 40 participants) and aimed to include a sufficient number of participants to explore recruitment and retention and to identify potential barriers in implementation. This approach is consistent with guidance that pilot studies should be large enough to estimate parameters for a future main study with reasonable precision, but not powered for effectiveness testing [56–60].

### Data collection and analysis

#### Qualitative data

Qualitative data were collected using FG with the participants [61]. The FG with the participants during the initial inpatient phase took place on-site at the clinics towards the end of the inpatient stay. The online FG took place via web conference when, at minimum, 2 weeks of the digital training phase had been completed. The focus group guides can be found in the supplement (see Supplement). The FG lasted between 70 and 98 min. The FG were conducted by the research team (J.G., J.S.), audiotaped, verbatim transcribed, and analyzed according to Mayring´s content analysis, using MAXQDA 2022 [62–64]. Transcripts were not returned to participants for comment and/or correction. Before data analysis, the research team (J.G., J.S., J.D.) became familiar with the interviews via the transcripts. Qualitative content analysis is a structured method based on predefined rules and research interests for analyzing qualitative data, ensuring consistency and intersubjectivity [63]. J.G. and J.S. coded the material. The category system was developed deductively beforehand based on the research questions. Additionally, categories were developed inductively from the material to ensure that all relevant themes were covered within the categories. The category system was rechecked by comparing it to the material, anchor examples, and research questions [65]. The category system can be found in the supplement (see Supplement). Analysis was discussed within the research team (J.G., J.S., A.S., J.D., and M.R.) and the working group for Qualitative Research at the Chair of Social Determinants of Health, ensuring rigor and integrity of the analysis. The analysis is framed by applying content-analytical quality criteria and reported using the COnsolidated criteria for REporting Qualitative research (COREQ) [54].

#### Quantitative data

Quantitative data collection occurred at the beginning of the intervention within the first 2 days of the inpatient phase (baseline/t1). Besides sociodemographic data, we included the following variables to characterize the intervention participants: the workability assessed using the German Work Ability Index (WAI) [66], quality of life using the German World Health Organization Quality of Life-BREF (WHOQOL-BREF) [67, 68], stress levels using the Depression Anxiety Stress Scale-21 (DASS-21) [69, 70], and eHealth Literacy (eHL) assessed with both the German version of the eHealth Literacy Scale (G-eHEALS) and the eHealth Literacy and Use Scale (eHLUS) [71–74]. An overview of the instruments used in the quantitative data collection (as well as information regarding the validation) can be found in the supplementary material (see Supplement). The newly developed eHealth Literacy Use Scale (eHLUS) can also be found in the supplementary material [74]. All were analyzed descriptively for sample characterization. Data analysis was conducted using the IBM SPSS software (Version 29.0.1.0). Descriptive statistics included means, standard deviations, ranges, and frequency distributions (percentages) to summarize continuous and categorical variables.

Quantitative and qualitative data are presented as merging integration results in a narrative discussion as well as displayed separately: the “Results” section shows firstly a side-by-side comparison discussion of quantitative and qualitative data results regarding the participants’ characteristics and, secondly, results regarding the acceptability, feasibility, and potentials for optimization according to the participants. In the “Discussion” section, both data analyses are displayed and discussed jointly, discussing convergences and divergences.

## Results

### Characterization of the participants

A total of 27 participants completed the intervention in the pilot study with a priori timeframe between October and December 2023. Twenty-one participants completed the web-based survey at the start of the inpatient phase (baseline data) between September and December 2023. In the qualitative data collection, 18 participants participated in a total of 4 FG between November and December 2023.

Table 1 presents the characteristics of the quantitative and qualitative study sample. The mean age is 53.8 years for the quantitative sample and 54.5 years for the qualitative sample. Regarding gender, the quantitative sample consists of 57.1% females, while the qualitative sample has an equal distribution of 50%. A total of 9.5% of the quantitative sample and 5.6% of the qualitative sample have a migration background. For sick days in the last 12 months (quantitative sample only), there is a wide range from 23.8% who had ≤ 15 days, 19.0% who had 16–30 days, 33.3% who had 31–60 days, 4.8% who had 61–90 days, and 19.0% who had ≥ 121 days.

**Table 1:** Participants’ characteristics of the quantitative (*n* = 21) and qualitative sample (*n* = 18)

Characteristics	Value of the quantitative sample	Value of the qualitative sample
Age in years mean (SD, min., max.)	53.8 (7.5, 34, 64)	54.5 (7.4, 34, 64)
Gender, (%)		
*Female*	57.1	50
*Male*	42.9	50
Migration background, (%)		
*Yes*	9.5	5.6
*No*	90.5	94.4
Sick days in the last 12 months, (%)		
≤ *15 days*	23.8	N.a
≥ *16–*≤ *30 days*	19.0	N.a
≥ *31–*≤ *60 days*	33.3	N.a
≥ *61–*≤ *90 days*	4.8	N.a
≥ *91–*≤ *120*	0.0	N.a
≥ *121 days*	19.0	N.a

Table 2 shows primary and secondary outcomes at the beginning of the inpatient phase (baseline data) to demonstrate the feasibility and appropriateness of the preventive intervention for the target group. The mean Work Ability Index (WAI) is 28 (*SD* = 4.6; range 21–38). Categorically, 47.6% have moderate workability, and 47.6% have poor workability, with no one having excellent workability. Quality of Life (WHOQOL-BREF) scores show means of 11.6 (*SD* = 1.3; range 9.7–13.7) for physical health, 12.7 (*SD* = 1.8; range 10–16.67) for psychological health, 14.5 (*SD* = 2.8; range 9.3–18.7) for social relationships, and 14.6 (*SD* = 1.8; range 11–18) for environmental health. Regarding mental health (DASS-21), 38.1% show symptoms of depression, 33.3% anxiety, and 47.6% stress. Health status is rated as good by 38.1%, fair by 57.1%, and poor by 4.8%, with no one rating it as excellent or very poor.

**Table 2:** Overview of outcomes at baseline (*n* = 21)

Outcome category	Value
Work Ability Index (WAI) mean, (SD, min, max)	28 (4.6, 21, 38)
Work Ability Index (WAI) as categorical representation, (%)	
*Excellent work ability*	0.0
*Good work ability*	4.8
*Moderate work ability*	47.6
*Poor work ability*	47.6
Quality of Life (WHOQOL-BREF), (SD, min, max)	
*Physical health*, mean	11.6 (1.3, 9.7, 13.7)
*Psychological health*, mean	12.7 (1.8, 10, 16.7)
*Social relationships*, mean	14.5 (2.8, 9.3, 18.7)
*Environmental health*, mean	14.6 (1.8, 11, 18)
Depression, Anxiety, and Stress (DASS-21), (%)	
*Symptoms of depression*	38.1
*Symptoms of anxiety*	33.3
*Symptoms of stress*	47.6
Health status (%)	
*Excellent*	0.0
*Good*	38.1
*Fair*	57.1
*Poor*	4.8
*Very poor*	0.0

In the FG, two main characteristics of the participants could be identified: firstly, the heterogeneity of the illness pattern (participants range from affective disorders, phobic and other anxiety disorders, adjustment disorders, and somatoform disorders to burnout) and, secondly, the stages of their illness, especially regarding the fact that the intervention aims at prevention.

The participants in the focus group perceived the clinical picture within the group as very heterogeneous as one of the participants stated as follows:

“But this clinical picture that we have here in the group is also very far apart.” (FG2, paragraph (p.) 72)

Regarding the aim and structure of the intervention as (secondary) prevention, participants stated difficulties arising regarding the different illness patterns. One participant experienced the intervention as “a tad too late in terms of my condition” (FG2, p. 43).

In contrast, participants with a less high burden perceived the intervention as a good fit on an individual as well as collective level as the following participant stated.

"So I also think, after these two weeks, almost everyone who is in the same position as we are now, i.e. not yet in burnout, but where there are the first signs, whether mental or physical, that it should really be offered to more people, so that perhaps some things don't even get as far as (name of another participant) said, that someone then takes the last step, right? So I think that could help, help a lot of people." (FG4, p. 58)

### Acceptability of the intervention

Intervention acceptability was explored via qualitative FG. Overall, the qualitative data show a great acceptance of the inpatient phase of the intervention.

"The inpatient phase there helped me a lot. I was able to switch off completely and I realized what I hadn't actually done in the years before. [...] So I rate these 14 days as a complete success." (FG4, p. 33)

Initially, participants were uncertain about the fact that recruitment for participation in the intervention was based on rejected rehabilitation applications. The applications submitted by the participant were rejected with the justification that there was no need for rehabilitation, and this intervention was proposed as a secondary prevention according to the status of the illness. This influenced the acceptance as well as the attitude towards participation in the intervention, as one participant stated that “[i]t has a bit of a flavor, it is just substitute satisfaction now” (FG3, p. 218). This initial uncertainty was, nonetheless, mostly resolved during the course of the inpatient phase. The intervention was compared to “summer camp” in the sense that “[a]t first you were afraid to go” then “you got used to it” and “didn't want to go home anymore” (FG4, p. 77).

The acceptance of the digital components of the intervention was optimistically cautious, albeit open in principle. At the same time, uncertainties concerning the individual use of the app could be identified, showing a broad spectrum about the acceptance of app use, as the following discussion in the focus group shows:

“P5: Yes, I do not mind the digital at all.

P1: Well, I am open to it too, I have to say, yes? That is the way it is in this day and age. You have to adapt a bit.

P5: Yes, the question is: (...) Is that enough for me? Probably not enough for me. (…)

P5: And will I really be able to cope with it? We do not know all that yet.

P1: No.

P5: We have not really worked intensively with it yet. That will not happen until we get home. [Agreement from other participants] And then there might be thousands more questions”

(FG2, p. 492-498)

This ambivalence was addressed again regarding the overall (normative) attitudes towards using the app and the prospective evaluation of the effects of the partially digital intervention. Statement ranged from being “a bit critical” with the demand to ask “again at the end of the twelve weeks” (FG1, p. 269) to being “cautiously optimistic” (FG1, p. 375).

### Feasibility of the intervention

The feasibility of the study procedures and data collection was confirmed, as quantitative data collection proved to be implementable and the response structures were plausible and as expected. This indicates that the applied instruments and the overall data collection process were suitable for the target group and the clinical setting.

In contrast, the feasibility of the intervention delivery was primarily explored through qualitative data. Participants’ feedback in focus groups provided insights into specific challenges and facilitators regarding the implementation and practical execution of both the inpatient and digital phases of the intervention. These qualitative findings informed the identification of barriers, optimization needs, and the perceived applicability of the intervention in everyday life.

#### Specifics of the inpatient phase

As already shown, the inpatient phase enabled the participants to take time out of everyday life, taking “time just for myself” (FG2, 394), breaking routines, and relaxing in a health-promoting manner. They especially emphasize that participants have time off and are not caught up in their daily routines.

The time of the inpatient phase, in combination with psychoeducation and the increased understanding of health, enabled the participants to reflect on and initiate behavioral changes. In addition, the participants reported a positive change in their experience of their condition, which enabled focus and reorganization of oneself, creating a meaningful experience.

"Apart from that, it was also a very, very welcome break for me. And I think it is also important that you cut off contact with the outside world a little bit, at least that is what I did for myself, that I really stopped contact with the outside world and really just concentrated on myself and my group, on what was important. And that really allowed me to switch off completely, sort myself out again, reset, question things that I usually did in everyday life, and/or so it was definitely a very important experience for me." (FG4, p. 32)

Participants indicated that the intervention created a (new) positive experience of their own condition. This experience was considered helpful in addressing that the participants are not alone with their illness and problems in everyday life, but that they can also recognize themselves in the experiences shared by other participants. This peer experience in “the crazy group here” (FG2, 394) also led to an intensive form of peer support during both the inpatient and digital training phases.

One of the main challenges of the inpatient phase was the expectations regarding the intervention as well as the pressure participants put on themselves, coupled with the existing burdens that the participants bring along. The participants were highly motivated for a change. However, this was countered by the deliberate therapeutic time-out and the change in pace at the start of the intervention. In summary, the following quote from a participant illustrates that the high motivation and the expectation towards the possible effect of the intervention for oneself meet the urgency and pressure of one’s own condition.

"[...] we all had in our heads: 'Yeah, man, we have got to get through this in just under two weeks, yeah, with which we can then get through the rest of our lives, I say, or afterwards, through everyday life, any techniques to avoid stress, etc.' But that was difficult for all of us and I think the time we needed to get used to [...] the pace (...) was perhaps something we missed a little, myself included, yes? o, the two or three days in the first week or so, I would have liked to have enjoyed them a bit more. Yes. We were always a bit, yeah: What's next?" (FG1, p. 125)

Another challenge was the organization and structure of the intervention. As the interventions start midweek on Thursdays, the organization of the participation in the intervention, as well as the travel and arrival in the clinic, was perceived as challenging. Mainly with participants who worked until Wednesday, the transition into the intervention and the overall “contrast” of the tempo was described as difficult as the following discussion in one of the FG shows:

“P3: And the contrast for all five of us who traveled there was that we all worked until Wednesday afternoon or evening, one hundred percent. (...)

P1: Yes.

P3: That put me under a bit of stress because I had a journey of over three hours, and then, of course, there were accidents and traffic jams in between. And then you get there, and you can't flip the switch.” (FG1, p. 146-150)

The transition from the inpatient phase to the digital training phase was also experienced to be challenging. On the one hand, there is now the expectation to apply the learned strategies and implement behavioral changes. On the other hand, the protected and intensive environment of the inpatient phase comes to an end. This concerns both the shift to “everyday life” and obligations such as work and care work that one is facing again, as described below by two participants.

“P1: (...) But for those who really need help here, there was a complete lack of time to get back to everyday life.

P5: So the thought of going home on Wednesday also stresses me out a bit. (...) Because I just do not know what is going to happen/ Then everything comes crashing down on me again.” (FG2, p. 424-425)

#### Specifics of the digital phase

Even though the transition to the digital training phase was perceived as challenging, it was emphasized that the support through the app and the ongoing therapeutic care are seen as beneficial. Participants reported that this makes them feel continuously supported and allows them to continue the work on themselves which was initiated during the inpatient phase. During the digital training phase, flexibility and low-threshold therapeutic contact were especially emphasized:

"Well, I think that is very good. [...] I think it has advantages. You do not have to drive. You do not have to sit down somewhere for hours. You have the therapist at home, so to speak." (FG1, p. 376)

The capacity and relationship building with the therapist gives the participants a feeling of security and continuity, which is well appreciated.

"I'm also very happy that I'm not home and alone after the 14 days, so to speak. I know that I can always call [name] if anything happens. [...] That gives me a bit of support and, well, a bit more courage, you could say" (FG1, p. 235–237)

The app is perceived as a mediator of the participants’ own condition and leads to a new perspective towards the participants’ own self and their health behavior on a continuous basis. The regular use of the app creates awareness of one’s own behavior. The possibility of using the app in their rhythm is especially well received. Modules are individually tailored between the therapists and the participants and are sent regularly on an individual basis which is also highlighted by participants. The mediating role of the app, as well as the individual approach of the app usage especially, leads to reflecting behavior as it is stated by the following participant:

"That [the content of the modules] makes you think. And I do not finish the modules or the module he sends me in an hour. I take my time with it. [...] I've familiarized myself well with this app. I also spend a lot of time with it." (FG1, p. 243)

As already stated, concerning the inpatient phase, the digital phase of the intervention shapes and affects the experience of one’s own condition, altering one’s own behavior, creating self-awareness, and creating possibilities of self-reflection.

Additionally, the role of peer-support during the digital outpatient phase is well emphasized by the participants. It seems to be crucial for motivation and adherence during the digital phase:

"And perhaps we need another reminder, someone to remind us of what we are doing and to think about ourselves. Because this app, you know, it is one thing, but we also have the group for this reason. The group acts as a reminder group for us: 'You need to stay on track. You must not fall back into old habits.'" (FG4, p. 130)

Regarding the use of the app in the digital outpatient phase, in some cases, an ambivalent relation with the app becomes apparent. The participants also described the app as an additional burden which is “tied to my leg too” and as “the next stress” (FG2, p. 634–635).

The need to use a new app also comes with uncertainty regarding one’s prerequisites and competencies. The continuous use of the app and the incorporation of the information and behavioral changes trained with the app are described as challenging.

"This is very, very difficult because we have learned that what we are doing in this app must transition into our daily lives. It must simply become a habit like making coffee and dusting." (FG2, p. 637)

This is why it is so important that the modules provided meet the needs of the participants and are also practicable in this respect, as the following quote illustrates exaggeratedly:

“Autogenic training on the train is also a bit bad. [Laughter]” (FG4, p. 90)

In the FG, the matter of eHealth Literacy among the participants was also addressed. Participants showed ambivalence regarding the use of the app, reflecting a spectrum of views from highly positive attitudes and high acceptance to more cautious or uncertain positions. Addressing the matter of competencies and prerequisites for using a digital application, one participant stated that it was necessary to acclimate to the app and to develop individual competency to deal with the app in a promising manner:

"You just have to try it and see for yourself: What is behind it? And once I have done it for an hour or two, I have an initial idea of where I am actually going. And what can I actually achieve? So, to assume that I'm overwhelmed by something is not going to get us anywhere. Perhaps we need to approach it with curiosity and say: 'Yes, this is my module, the best one for me personally, based on my situation.' And then I just look at what's in there. And then I pick out what I need." (FG2, p. 648)

While some participants stated that they feel able to use the app, some stated that they are insecure regarding the use of the app as they merely use apps on their smartphone elsewhere. Illustratively, one participant positively compared the use of the app to “looking on Ebay or checking Facebook” so that “you can also open this app and do something for yourself with the app” (FG4, p. 87). Contrastively, another participant negatively highlighted that “[y]ou do not usually work with an app” (FG2, p. 828–830), and that apps are no part of everyday life.

This range of different levels of eHealth Literacy can be confirmed within the quantitative data. In addition to the sociodemographic variables and outcomes, eHealth Literacy was assessed using the German eHEALS, which allows a total score of between 8 and 40 points, and a self-developed and validated instrument for context-specific eHealth literacy (eHLUS), which allows a total score of between 14 and 70 points. Table 3 shows the participants’ eHealth Literacy scores. A higher score on both general and context-specific instruments indicates a higher level of eHealth literacy. The results show an overall moderate score with a difference in minimum and maximum in both the general and context-specific eHL (see Table 3).

**Table 3:** Overview of eHealth Literacy (*n* = 21)

eHL	Value
General eHL (eHEALS, eight items) mean, (SD, min, max)	29.2 (6.3, 14, 37)
Context-specific eHL (eHLUS, 14 items), (SD, min, max)	51.1 (7.7, 33, 66)

#### Themes identified for the further development of the intervention

Overall, feasibility is given, but challenges have been identified. Additionally, several aspects regarding the further development of the intervention were stated in the focus groups. Firstly, individual, therapeutic, and communal aspects such as the anticipation of the high motivation of the participants, the purposeful introduction and embedment of the app within the therapeutic support, the different actors and roles within the intervention, the relationship with the therapists, and the peer support can be presented. Secondly, organizational and structural aspects occurred, such as the period of the intervention and transition into the inpatient phase, the possibility of evaluation within and between the peer group at the end of the digital phase, the app usage beyond the duration of the intervention, and the communication with employers.

**Table 4:** Overview of aspects for further development of the intervention

Domain	Theme	Description	Anchor examples
**Individual, therapeutic, and communal aspects**	*High motivation of the participants*	Participants are highly motivated to work intensively during the 2 weeks due to their distress. This contrasts with the deliberately set break and slow pace at the beginning of the intervention. Participants expressed that this time needs to be communicated more purposefully, and that ways should be found to utilize this time more effectively (e.g., with orientations and organizational tasks)	“P2: But I thought the weekend we had, (…), was actually not bad, as we could relax. But Thursday, it was, yeah, it was too hectic for us too P3: Yes. Yes, yes P2: Because we knew nothing, right? We were curious. We were excited and suddenly we were alone P1: We actually wanted to get started. We wanted to begin” (FG1, p. 155–158)
	*Introduction to the app, the specific modules, how to use the app, and how the app is embedded within the therapeutic support*	Of central importance to the participants was a targeted and content-rich introduction to the app during the inpatient phase. The app should be presented in its entirety, showcasing its content and functional features comprehensively. An early insight into the structure and usage of the app, ideally along specific modules, should be provided. Additionally, it was emphasized that it is crucial to clarify and provide detailed information about the everyday use of the app and the necessary resources beforehand. Overall, it was emphasized that the objectives of using the app and how it is integrated into therapeutic support should be communicated clearly	“But how long do I need for one module? Do I need a whole week? Do I need two hours? How long do I need at all? These are also questions that pop into my head” (FG2, p. 637) “Or maybe not just getting all the modules at the end, I mean, we already have the module 'Goals' and 'Relaxation', that's all good and well. I could theoretically download that somewhere from YouTube, but I need the app to do something completely different with myself, like dealing with depression, burnout, or emotions, who knows what else, so that you integrate that from the beginning to know: What does such a complex module actually look like?” (FG2, p. 630)
	*Interweaving the different actors and roles within the intervention*	It was important for the participants to clarify the role of the therapists and that of the group, as well as to clearly define expectations and potential roles of each individual actor. While therapists maintain continuous contact, providing substantive input and allocating modules, their role primarily involves giving feedback and offering alternative perspectives on completed tasks and behavioral changes. The group, on the other hand, serves not only for emotional and technical ad hoc support but is also regarded as part of the therapeutic process	“And I could imagine that therapists could also provide a different idea or another push, right? That one could look at things from the other side again. But for example, since we don't have much contact with these therapists, I also see the group as somewhat therapeutic, don't I?” (FG4, p. 123)
	*Relationship with the therapists and integration of the app into the therapeutic relationship*	The central role of therapists is intended to continue for participants even during the digital phase. The app serves as a support in this regard and makes an important contribution throughout the intervention. The modules that participants work on should be integrated into the therapeutic relationship and, if possible and necessary, discussed and re-evaluated in conversations with therapists	“Well, you're supposed to have a therapist in the background of this app, right? They're supposed to give you the answers. It shouldn't just be like reading a book, right? Or where you're just having monologues and you can only read it. It should be a personal reaction, shouldn't it?” (FG3, p. 232) “Well, basically, the things we've written in there [the app], she could just maybe review them and give us feedback, maybe see things differently, tackle them differently, or just give us a tip again. So that's my expectation, right? I mean, you have your own idea, but is it always the right one, or maybe are you getting lost in the wrong direction?” (FG4, p. 123)
	*Fostering peer-support*	The participants emphasized the importance of the group both during the inpatient phase and the digital training phase. In this regard, there was repeated desire to strengthen the group dynamics as part of the intervention and to enhance the role of the group in the therapeutic process. Concrete optimization needs included the possibility of a group chat function within the app	“P1: We've also mentioned before, maybe we could create a WhatsApp group within the app. That might not be a bad idea. Where everyone can share their thoughts like: 'Do you understand this? What does that mean? Am I interpreting this incorrectly?' Because there are always multiple meanings to a word, right? And then, maybe we could add something like (…) P5: Yes, that could be another good idea. A group, just like we are now, through this app P8: Maybe bundling that in the app, the group that was together here.” (FG2, p. 648–649)
**Organizational and structural aspects**	*Period of the intervention and transition into the inpatient phase*	Participants have suggested starting the intervention on Monday. This way, preparation and travel could take place over the weekend, making the transition smoother, as participants would not be tied up with work until the day before the start of the inpatient phase. Besides this organizational aspect, it is also emphasized that this would allow participants to mentally prepare differently for the intervention: the first week could be dedicated to settling in, taking a break, and trying out the modules, while the second week could be used for more intensive work and preparation for the digital phase	“And it's also more pleasant when you're driving if the roads are less busy. No, that's/Yes, that's just a suggestion, because you’re immediately back into everyday life and you still have the weekend with your family or at least Sunday.” (FG3, p. 277) “I would also support arriving on Monday and having the option to say until Friday, 'I'll do this and that.' And then being able to decide for oneself about the second week.” (FG2, p. 340–342)
	*Wish for evaluation within and between the peer group*	In addition to the regular individual coaching sessions with the therapists and the concluding session at the end of the intervention, participants could find value in an evaluation within the group. During such a session, participants could share their experiences and develop concrete strategies for implementing the learned techniques beyond the interventions’ end and in the long term. Participants could imagine a joint online meeting (comparisons were made with the focus groups conducted) or an inpatient refresher day, perhaps also an additional stay in the clinic for a few days	“And evaluating, now, let's say, in an online meeting like just now (…), and when you do it inpatient, they're all there again and, as we say in German, ‘can't escape’. And that was an idea. Of course, we're aware that all of this is associated with costs, if nine people were to be treated inpatient again, but we all see that as positive, or would see it as positive, if that were to happen again. And then you could exchange ideas: How did it work in everyday life? Because that's the crucial part. How can you integrate the whole thing into everyday life and how do you implement it?” (FG4, p. 114)
	*Continued app usage beyond the duration of the intervention*	Participants expressed a desire for the option to continue using the app beyond the digital training phase, even without the integration into therapeutic support	“P6: The twelve weeks won't be enough P5: No P6: I guess some will say, ‘I have to keep going back to it.’” (FG2, p. 772–774)
	*Information letter for employers*	Participants wished for a separate document to present to their employers regarding their participation in the intervention, containing as little information as possible and as much as necessary	“And maybe just another separate letter with the important details, like when I'll be away from work, without all the other information that's relevant to me. That might be another option.” (FG2, p. 171)

Table 4 gives a synthesis of these possible aspects along with an anchor example of the qualitative data.

## Discussion

### Principal results

The aim of this piloting study was to provide a better understanding of the target group of the intervention and enable important insights regarding chances, challenges, and specifics of the implementation of the new intervention. While the study explored the characteristics of participants who took part in the pilot, this should not be understood as reaching a population outside the intended target group. In accordance with the diagnostic and indication areas defined for the intervention, the participants matched the target population. However, given that both the intervention and the recruitment pathway were newly developed and had not yet been systematically evaluated, we deliberately adopted an exploratory perspective to examine who would engage with the program. This approach allowed us to remain open and sensitive to potential heterogeneity in participants’ symptom profiles and burden levels. Such heterogeneity is particularly relevant for a newly designed preventive intervention, as it provides valuable insights for refining future recruitment and tailoring the program to diverse participant needs. The heterogeneity of the participants is emphasized in both the quantitative and qualitative data and refers to differences in sociodemography, individual life histories, overall health status, sick days in the last 12 months and symptom burden, and many other factors. As this pilot study was designed as an exploratory study, no fixed progression criteria were predefined. Instead, the collected data served to identify indicators for a future evaluation phase. Qualitative analysis supported these findings, with several participants describing the digital phase as a meaningful support, although some reported challenges in using the app.

Several aspects regarding adjustments and possible optimizations of the intervention were mentioned. The results indicate a generally high acceptance of the intervention, though challenges in app usage were also observed. The defined feasibility criteria were narrowly met, highlighting the need for further optimization steps. In particular, the main study should focus on expanding support options for app usage to enhance adherence and user experience. Overall, the results of the study showed an ambivalence of the usage of the app which can be addressed within the intervention tackling the mentioned challenges of the participants through adjustments during the intervention and therapeutic support. In a didactical manner, it was of great importance to have insights regarding the transition back into everyday life as well as the challenging integration of the app usage in everyday life. The essential meaning of the group and the peer support in both, the inpatient phase and the digital outpatient phase, was emphasized by the patients. Group cohesion is a well-known factor especially in mental health care [75–78]. Another key element was the attitudes towards the app as well as the varying eHealth Literacy of the participants which also need to be integrated within the therapeutic relationship with the participants to adequately deal with the different levels of health literacy, prerequisites, competencies, and insecurities of the participants regarding the app usage. In addition, the study highlights the specifics of the combination of an inpatient phase with an app-supported digital outpatient phase, filling an important gap regarding the design, conduct, and evaluation of an intervention combining both an inpatient and outpatient setting.

Recognizing the various determinants of the use of digital health applications like digital health literacy, cultural and social factors, and the motivation, this study confirms the complexity of designing, implementing, and conducting a digital intervention [26, 79–81]. Regarding the adherence and the engagement towards digital health interventions, our study also shows an ambivalence and various challenges as it is also shown by other studies [82–84]. Overall, considering the heterogeneity of the participants seems to be of importance for the intervention. The influence of the heterogeneity/diversity of participants and varying illness patterns for the effectiveness of preventive measures as well as the use of digital health applications is also shown by other studies [21, 22]. The literature also emphasizes the importance of considering social determinants of health and the heterogeneity of target groups in the development and implementation of preventive measures in the field of mental health [85, 86]. Beside the various determinants regarding the individual use of the app, the participants addressed a variety of organizational and contextual factors such as the timing of the intervention as well as the transition into the intervention and back to everyday life. The meaning of accounting and evaluating the concrete health care setting where the intervention will be deployed is emphasized in the literature [28, 33, 87, 88]. Only through tailored approaches that take into account the diversity of needs and backgrounds it is possible to meet the varying demands and promote health behavior [86]. Acknowledging the heterogeneity of the participants regarding sociodemographic characteristics, outcomes, and the subjectively experienced broad range of health status, the overall acceptability and feasibility as well as the challenges and chances of the intervention the results of the piloting do not indicate great differences. Subsequently, those differences need to be addressed in detail within the further evaluation of the intervention as there is evidence of a relationship between digital health literacy and adherence of digital health interventions [82, 83].

The results of our study align with prior evidence that underscores both the promise and persistent challenges of delivering digital mental health interventions, especially with respect to engagement, adherence, and integration into routine care[89]. For instance, Lipschitz et al. [90] in their review of digital mental health interventions highlight that sustained engagement remains a central obstacle, often affected by design features, user burden, and contextual fit [90]. Löchner et al. (2025) likewise note that while digital technologies hold transformative potential, their successful adoption depends heavily on embedding them into existing care pathways and workflows and not as standalone tools [91]. At the same time, our findings add to this literature by providing insights from a preventive, app-supported program embedded in a clinical rehabilitation context, which remains a relatively underexplored setting in digital mental health research. In such settings, unique challenges may arise in aligning digital intervention use with existing care pathways, workflow constraints, and patient expectations as it is shown that engagement rates in digital mental health interventions vary greatly across settings, and conceptual interpretations of engagement remain inconsistent [92]. In addition, a review of mental health and health behavior change interventions underscores that context, user characteristics, and setting moderate engagement and behavior change effects [93]. Overall, the challenges of aligning user expectations, clinical workflows, and digital health usability may be more complex than in stand-alone or purely outpatient digital programs [94–96]. Our pilot study results suggest important aspects about how a blended care format including inpatient and digital outpatient phases affect engagement trajectories, adherence and dropouts, and user satisfaction in a rehabilitation environment.

### Implications for the upcoming evaluation phase

The upcoming evaluation phase will build on the insights gained from this pilot study to analyze the implementation and effectiveness of the psychosocial prevention intervention in a larger sample [23]. Pilot studies play a crucial role in digital health research, as they help identify usability challenges, technical issues, and implementation barriers before scaling [97, 98]. They also provide essential insights into acceptability and engagement patterns, which are key determinants of successful adoption and integration into routine care [99, 100]. Implementation frameworks suggest that without such early insights, digital interventions often fail at the adoption stage or face contextual barriers [100]. The key research questions in the upcoming larger study are as follows: (1) Has the implementation of the intervention been successfully achieved? and (2) Is the intervention effective in achieving its intended outcomes? To address these questions, in the qualitative research stream of our mixed-methods design, we will examine the organizational conditions, accessibility of the target population, and the collaboration among project partners involved in the implementation. This analysis will incorporate perspectives from both professionals and intervention participants via expert interviews as well as focus groups. The research will therefore adapt and focus on the aspects mentioned in Table 4 for further improvement of the intervention. Moreover, transparency and accessibility of recruitment pathways will remain an important focus in the upcoming evaluation phase. In this next step, additional access pathways will be included, such as through health insurance providers and public outreach activities of the participating clinics. Beyond these structural aspects, the question of who actually engages with the intervention will also remain central. Examining the characteristics of participants recruited through different pathways will provide valuable insights into reach and representativeness and help to further refine the targeting and delivery of the intervention. Studies in digital health and health promotion have shown that different recruitment strategies vary markedly in cost, reach, and demographic bias, and that retention and generalizability may depend on how participants were recruited [89, 101–104].

Furthermore, we will assess the intervention’s overall effectiveness. We will perform a pre-post design effectiveness assessment. The primary outcomes of interest include workability, measured using the German Work Ability Index [66], and quality of life, assessed with the German WHO Quality of Life-BREF [67, 68]. Stress levels will be evaluated using the Depression Anxiety Stress Scale-21 (DASS-21) [69, 70], and eHealth literacy will be measured using the G-eHEALS and a self-developed instrument designed to assess holistic digital health literacy (DHL) [71–73, 105]. Secondary outcomes include anxiety and depressive symptoms (DASS-21) and self-efficacy, evaluated using the SWOP-K9 [106, 107]. Detailed information on the validity and reliability of these assessment instruments is available in the Supplement.

Behavioral data from the training phase which will contrast the qualitative data regarding acceptability presented in this study will be analyzed according to the FITT criteria (frequency, intensity, time, and type), allowing us to assess participants’ adherence to the eHealth application [89, 108, 109].

### Limitations

There are several limitations to this study. The study focused on the characterization of the target group of the intervention, the acceptability, and feasibility of the app-supported intervention regarding its specifics and challenges as well as possible adjustments and optimization of the intervention. Although the quantitative sample was limited, the collected data provide valuable insights into the feasibility of the intervention. A limitation of this study is that no pre-post assessment was conducted. This decision was deliberate, as the pilot study primarily aimed to examine the feasibility and implementation of the newly developed intervention rather than evaluate its effectiveness. Given the small sample size, a pre-post design would not have provided sufficient statistical power or meaningful interpretability. Instead, the focus of this phase was placed on qualitative exploration, baseline assessments of participants’ burden profiles, and testing the feasibility of both data collection and the intervention itself. The evaluation of effectiveness is therefore planned for the subsequent, larger-scale evaluation phase of the project. Due to the lack of quantitative analysis of the feasibility, it was not possible to compare the intervention regarding acceptability and satisfaction, as it is broadly discussed in literature [84].

Additionally, the timing of the conducted FG differs as two took place during the inpatient phase and two took place during the digital phase. In that way, it was possible to get insights into both phases of the intervention enabling a holistic view of the participants’ view on the intervention which was a great benefit for evaluating the piloting. For evaluating the intervention in an adequate manner with qualitative data in the upcoming evaluation phase of the project, focus groups will be coherently conducted when at least 8 weeks of the digital phase have passed. This relates to the aspect that the course of the intervention as well as the combination of the inpatient and digital phase need to be evaluated on the basis that participants have sufficient experience with the intervention. This also accounts for the fact that the integration of the behavioral changes and the usage of the app as well as the transition back to everyday life is experienced as challenging by the participants. The findings regarding the acceptability related to differentiating which individuals experienced the most effect, success, and motivation with the intervention also need to be confirmed within a larger qualitative sample in the upcoming evaluation phase.

## Conclusions

Work-related health issues are an increasing burden in the population and are affecting health systems in a great manner. A prevention intervention addressing early burdens and symptoms may offer great potential. Combining both an intensive supporting inpatient phase and an app-supported digital outpatient phase may contribute to strengthening participation in working life. The results of this study provide the first promising insights regarding the newly developed prevention intervention “RV Fit Mental Health” addressing both chances and challenges. The results show an acceptance of the intervention and name aspects for further optimization of the intervention. Nonetheless, the results warrant various adaptations and further evaluation within a larger trial especially focusing on the efficiency of the intervention. Also, didactical optimization within the therapeutic support is required. If the future results of the evaluation phase prove the efficacy of the intervention and the results are accompanied by successful implementation, the approach of the intervention “RV Fit Mental Health” could be a promising and purposeful intervention addressing mental health-related issues and strengthening participation in working life.

## Supplementary Information

Additional file 1: Interview guide of the focus groups.

Additional file 2: Category system.

Additional file 3: Description of Measurements Instruments.

Additional file 4: COREQ Reporting Checklist.

Additional file 5: CONSORT 2010 checklist of information to include when reporting a pilot or feasibility trial.

## Data Availability

Data are available from the corresponding author upon reasonable request.

## Abbreviations

AOK PLUS: Allgemeine Ortskrankenkasse PLUS
CONSORT: Consolidated Standards of Reporting Trials
COREQ: COnsolidated criteria for REporting Qualitative research
DASS-21: Depression Anxiety Stress Scale-21
eHEALS: EHealth Literacy Scale
eHL: EHealth Literacy
eHLUS: EHealth Literacy and Use Scale
FG: Focus group
G-eHEALS: German version of the eHealth Literacy Scale
Max.: Maximum
Min.: Minimum
P: Paragraph (within the transcript)
PE^3^PP: German acronym for the project for the Development, piloting, and evaluation of an app-supported psychosocial prevention intervention
SD: Standard deviation
TUM: Technical University of Munich
WAI: Work Ability Index
WHOQOL-BREF: World Health Organization Quality of Life-BREF
