# Supplementary material for: Pilot study of an app-supported psychosocial prevention intervention: a mixed-methods approach
Source: Pilot Feasibility Stud. 2025 Dec 1;11:155. doi: 10.1186/s40814-025-01737-y (PMC12670738; doi:10.1186/s40814-025-01737-y)
Supplement: Supplementary file 1 — Additional file 1: Interview guide of the focus groups. [file 40814_2025_1737_MOESM1_ESM.docx]

*Additional File: Interview guide of the focus groups*

1. **Focus group guide inpatient phase (english translation)**

Research questions of the focus groups

1. What are the experiences, expectations and needs of the first cohorts of participants when going through the prevention intervention?
2. From the participants´ point of view, are there ways in which the prevention intervention can be optimized under the given conditions?

Sample of the focus group

1. Participants undergoing the prevention intervention (currently in the inpatient phase)

Main themes of the focus groups

1. Participants´ experiences with the parts of the intervention completed to date
2. Demands and needs for the prevention intervention
3. Expectation, potentials and challenges

**In advance:**

- Consent forms
- Remarks on the focus group process
- Discussion rules and clarification of the moderator´s role
- Information on the start of the recording

| **Key questions** | **Notes and remarks** |
| --- | --- |
| Introduction round (moderators first) |  |
| Key questions on main theme 1:   1. Now that you are in the middle of the inpatient phase, what are your experiences overall? |  |
| Key questions on main theme 2:   1. Why did you decide to take part in the prevention intervention? What were your demands? 2. What are your expectations and wishes with regard to the prevention intervention? |  |
| Key questions on main theme 3:   1. How do you feel about the digital training phase? How do you feel about using the app? 2. What challenges are you expecting to face in integrating the prevention intervention into your everday life? 3. What do you think it will take for you to benefit from the prevention intervention even after completing the subsequent digital training phase? |  |
| Summary of the discussion by moderators (with the possibility of additions and comments by the participants) |  |
| Flash with participants: How was the focus groups? Were there any insights? |  |

**Afterwards:**

- Socio-demographic questionnaire
- Consent forms
- Thank you and outlook

1. **Focus group guide digital phase (english translation)**

Research questions of the focus groups

1. What are the experiences, expectations and needs of the first cohorts of participants when going through the prevention intervention?
2. From the participants´ point of view, are there ways in which the prevention intervention can be optimized under the given conditions?

Sample of the focus group

1. Participants undergoing the prevention intervention (currently in the digital outpatient phase)

Main themes of the focus groups

1. Participants´ experiences with the parts of the intervention completed to date
2. Demands and needs for the prevention intervention
3. Expectation, potentials and challenges

**In advance:**

- Consent forms
- Remarks on the focus group process
- Brief introduction to Zoom and the interface - Use of chat and emergency number information
- Reference to camera
- Discussion rules and clarification of the moderator's role
- Note on start of recording and the additional computer dialed in for recording

| **Key questions** | **Notes and remarks** |
| --- | --- |
| Introduction round (moderators first) |  |
| Key questions on main theme 1:   1. Now that you have already gone through the inpatient phase and a large part of the digital training phase, what are your experiences overall? |  |
| Key questions on main theme 2:   1. Why did you decide to take part in the prevention intervention? What were your demands? 2. What are your expectations and wishes with regard to the prevention intervention? What did you like? Did you miss anything? |  |
| Key questions on main theme 3:   1. How was the transition into the digital outpatient phase? What are your experiences so far? How do you feel about using the app? 2. How did you manage to integrate the prevention content into your everyday life? What challenges do you face in integrating the prevention content into your everyday life? 3. What is the value of the monthly therapist meetings and the network with the group? 4. What do you think it will take for you to benefit from the prevention intervention even after completing the subsequent digital training phase? |  |
| Summary of the discussion by moderators (with the possibility of additions and comments by the participants) |  |
| Flash with participants: How was the focus groups? Were there any insights? |  |

**Afterwards**

- Socio-demographic questionnaire
- Consent forms
- Thank you and outlook
