# Supplementary material for: Pilot study of an app-supported psychosocial prevention intervention: a mixed-methods approach
Source: Pilot Feasibility Stud. 2025 Dec 1;11:155. doi: 10.1186/s40814-025-01737-y (PMC12670738; doi:10.1186/s40814-025-01737-y)
Supplement: Supplementary file 2 — Additional file 2: Category system. [file 40814_2025_1737_MOESM2_ESM.docx]

Additional File: Category system

| **Category** | **Subcategory** |
| --- | --- |
| Target group of the intervention | Subjective clinical picture and experience of illness  Preventive character  Pictorial description  Remarks on socio-demographic data |
| Motivation | Symptoms as motivation  Lifestyle change  Rehabilitation vs. prevention  External Motivation  Decision-making process for participation in intervention |
| Experiences, critic and feedback | Positive feedback  Criticsm of intervention  Criticsm of DRV  Ciriticsm of the clinics  Adjustments and Optimization |
| Expectations, wishes and demands | Uncertainties and insecurities  Expectation towards the intervention  Expectation of oneself |
| Inpatient phase | Chances and Potentials  Challenges  Acceptance  Introduction of the app |
| Digital outpatient phase | Chances and Potentials  Challenges  Acceptance  App usage  Everyday integration  Health Literacy  Role of the therapists |
| Effects of the intervention | Time off and pattern change  Group dynamic and peer support  Self-efficacy and empowerment  Behavioral change |
| Dissemination of evaluation results | - |
