# Supplementary material for: Pilot study of an app-supported psychosocial prevention intervention: a mixed-methods approach
Source: Pilot Feasibility Stud. 2025 Dec 1;11:155. doi: 10.1186/s40814-025-01737-y (PMC12670738; doi:10.1186/s40814-025-01737-y)
Supplement: Supplementary file 3 — Additional file 3: Description of Measurements Instruments. [file 40814_2025_1737_MOESM3_ESM.docx]

Additional File: Description of Measurements Instruments

| **Instrument Name** | **Measurement** | **Number of Items** | **Abbreviation** | **Validity and Reliability** | **Calculation** | **Interpretation** | **Source** |
| --- | --- | --- | --- | --- | --- | --- | --- |
| Depression Anxiety Stress Scale-21 | Depression, anxiety, stress | 21 | DASS-21 | Internal consistency: Depression α=0.94, Anxiety α=0.87, Stress α=0.91 | Sum of items for each subscale. Highest score: 63, lowest score: 0. | Higher scores indicate more severe symptoms. Categorization: Normal to extremely severe, based on cutoff scores. | Antony MM, Bieling PJ, Cox BJ, et al. Psychometric properties of the 42-item and 21-item versions of the depression anxiety stress scales in clinical groups and a community sample. Psychological Assessment 1998;10:176–81.  Nilges P, Essau C. Die depressions-Angst-stress-Skalen: der DASS- -ein Screeningverfahren Nicht Nur Für Schmerzpatienten. Schmerz 2015;29:649–57 |
| eHealth Literacy Scale | Ability to find, evaluate, and apply health information from the internet | 8 | eHEALS | Internal consistency: α=0.88 (youth), α=0.94 (adults) | Sum of items. Highest score: 40, lowest score: 8. | Higher scores indicate higher eHealth literacy. | Norman CD, Skinner HA. eHEALS: the eHealth literacy scale. J Med Internet Res 2006;8:e27.  Chung S-Y, Nahm E-S. Testing Reliability and validity of the eHealth literacy scale (eHEALS) for older adults recruited online. Comput Inform Nurs 2015;33:150–6.  Soellner R, Huber S, Reder M. The concept of eHealth literacy and its measurement. J Media Psychol 2014;26:29–38. |
| eHealth Literacy and Use Scale | Autonomous use of medical apps, eHealth engagement, eHealth literacy, technical acces | 14 | eHLUS | Internal consistency: α=0.93 | Sum of items. Highest score: 70, lowest score: 14. | Higher scores indicate higher eHealth literacy in the context of using medical apps. | Preprint: Stephan, J., Gehrmann, J., Stullich, A., Dehner, J. & Richter, M. Development and Validation of the eHealth Literacy and Use Scale (eHLUS) to Measure Medical App Literacy; 10.21203/rs.3.rs-4377708/v1 (2024). |
| MacArthur Scale of Subjective Social Status | Perception of one's own social status relative to others | 1 | MacArthur SSS Scale | Moderate test-retest reliability (ρ = 0.47 within one week, ρ = 0.32 after one year). Fair agreement with WAMI index (Kappa = 0.26). | Single item scored from 1 to 10. | Higher scores indicate higher perceived social status. | Amir, D., Valeggia, C., Srinivasan, M., Sugiyama, L. S. & Dunham, Y. Measuring subjective social status in children of diverse societies. PloS one 14, e0226550; 10.1371/journal.pone.0226550 (2019).  Zhao, M. et al. Subjective socioeconomic status: an alternative to objective socioeconomic status. BMC medical research methodology 23, 73; 10.1186/s12874-023-01890-z (2023). |
| WHO Quality of Life Instrument, Short Form | Physical health, psychological health, social relationships, environment | 26 | WHOQOL-BREF | Internal consistency: Physical health α=0.88, Psychological health α=0.83, Social relationships α=0.76, Environment α=0.78 | Average scores for each subscale. Physical health: 7-35, Psychological health: 6-30, Social relationships: 4-20, Environment: 8-40. | Higher scores indicate better quality of life. | Skevington SM, Lotfy M, O’Connell KA, et al. The world health organization’s WHOQOL-BREF quality of life assessment: Psychometric properties and results of the International field trial. A report from the WHOQOL group. Qual Life Res 2004;13:299–310. |
| Work Ability Index | Ability to work, physical and mental health, job requirements, job satisfaction | 7 | WAI | Internal consistency: α=0.75 | Weighted sum of items. Highest score: 49, lowest score: 7. Categories: Poor (7-27), Moderate (28-36), Good (37-43), Excellent (44-49). | Higher scores indicate better work ability. Categorization: Poor, moderate, good, excellent based on cutoff scores. | Freyer M. Eine Konstruktvalidierung des work ability index Anhand Einer Repräsentativen Stichprobe von Erwerbstätigen in Deutschland: Bundesanstalt Für Arbeitsschutz und Arbeitsmedizin (Baua). 2019. |
