# Supplementary material for: Pilot study of an app-supported psychosocial prevention intervention: a mixed-methods approach
Source: Pilot Feasibility Stud. 2025 Dec 1;11:155. doi: 10.1186/s40814-025-01737-y (PMC12670738; doi:10.1186/s40814-025-01737-y)
Supplement: Supplementary file 4 — Additional file 4: COREQ Reporting Checklist. [file 40814_2025_1737_MOESM4_ESM.docx]

Additional File: COREQ Reporting Checklist

| Domain 1: Research team and reflexivity | |
| --- | --- |
| Personal Characteristics | |
| 1. Interviewer/facilitator | Detailed in methods section "Data collection":  Interviewer: Jan Gehrmann (JG), Johannes Stephan (JS) |
| 1. Credentials | JG: M.A.  JS: M.Sc.PH, M.Sc.  Jana Dehner (JD): B.Sc.  Ananda Stullich (AS): M.A.  Matthias Richter (MR): Prof. Dr. rer. soc. |
| 1. Occupation | JG, JS, AS: research associates  JD: Student assistant  MR: Professor (Chair of Social Determinants of Health at TUM), project coordinator |
| 1. Gender | JG, JS, MR: Male  JD, AS: Female |
| 1. Experience and training | Interviewers had significant experience in qualitative research and were led by an experienced researcher (MR). Additionally, they attended external workshops and informal training.  Experience:  JG: Significant experience in qualitative research and interviews, realized qualitative interviews  JS: significant experience in qualitative research and interviews, realized qualitative interviews  JD: experience in qualitative research and interviews  AS: significant experience in qualitative research and interviews  MR: conceptualized and realized several qualitative interview studies previously |
| Relationship with participants | |
| 1. Relationship established | The authors who collected the data or analysed them (JG, JS, JD, AS) had no prior existing relationship with the interviewees. |
| 1. Participant knowledge of the interviewer | Interviewees were informed about the project, interviewer’s educational background and occupational status in advance. Participants had the chance to request further information regarding the provided information. |
| 1. Interviewer Characteristics | The interviewers have a research interest in health services research and (medical) sociology. |
| Domain 2: Study design | |
| Theoretical Framework | |
| 1. Methodological orientation and theory | Reported in the methods section "Analysis". |
| Participant selection | |
| 1. Sampling | Reported in the methods section. |
| 1. Method of approach | Reported in the methods section. |
| 1. Sample size | Reported in the methods section. |
| 1. Non-participation | Reported in the methods section. |
| Setting | |
| 1. Setting of data collection | Reported in the methods section "Data collection and analysis":  Interviews: professionals’ workplaces or at home.  Additional information: Interviewees attended the focus groups either in the clinics or digitally at home. |
| 1. Presence of non-participants | No one else was present besides the participants and the interviewer. In the digital data collection, in some cases, non-participants were present at the beginning to provide technical support. |
| 1. Description of Sample | Reported in the methods section. |
| Data Collection | |
| 1. Interview Guideline | The focus group guides are described in the methods section "Data collection and analysis" and can be found in the Additional Files. |
| 1. Repeat interviews | No repeat interview was necessary. |
| 1. Audio/Visual recording | Reported in the methods section "Data collection and analysis". |
| 1. Field notes | No field notes were written. |
| 1. Duration | Reported in the methods section. Focus groups lasted between 70 and 98 minutes. |
| 1. Data saturation | Reported in the methods section "Data collection and analysis". |
| 1. Transcripts returned | Transcripts were not returned to participants. |
| Domain 3: Analysis and findings | |
| Data analysis | |
| 1. Number of data coders | Reported in the methods section "Data collection and analysis":  Indexing all interviews and analysing: JG, JS, JD  Summarizing and charting of the indexed data: JG, JS, JD, AS |
| 1. Description of the coding tree | Reported in the methods section "Data collection and analysis". The category system can be found in the Additional Files. |
| 1. Derivation of themes | Reported in the methods section "Data collection and analysis". |
| 1. Software | Reported in the methods section "Data collection and analysis". |
| 1. Participant checking | Not reported. |
| Reporting | |
| 1. Quotations presented | Different participants' quotes are presented in the results to illustrate the findings, and a number identifies each quotation. |
| 1. Data and findings consistent | Yes. |
| 1. Clarity of major themes | Major themes are presented in the results. |
| 1. Clarity of minor themes | As far as the word count permits, we discuss minor themes, too. |
